# Seed traits matter—Endozoochoric dispersal through a pervasive mobile linker

**DOI:** 10.1002/ece3.8440

**Published:** 2021-12-14

**Authors:** Jonas Stiegler, Katrin Kiemel, Jana Eccard, Christina Fischer, Robert Hering, Sylvia Ortmann, Lea Strigl, Ralph Tiedemann, Wiebke Ullmann, Niels Blaum

**Affiliations:** ^1^ Institute of Biochemistry and Biology, Plant Ecology and Nature Conservation University of Potsdam Potsdam Germany; ^2^ Institute of Biochemistry and Biology Evolutionary Biology / Systematic Zoology University of Potsdam Potsdam Germany; ^3^ Institute of Biochemistry and Biology, Animal Ecology University of Potsdam Potsdam Germany; ^4^ Department of Agriculture, Ecotrophology, and Landscape Development Faunistics and Wildlife Conservation Anhalt University of Applied Sciences Bernburg Germany; ^5^ Leibniz Institute for Zoo and Wildlife Research (IZW) Berlin Germany

**Keywords:** agricultural landscapes, endozoochory, *Lepus europaeus*, mobile links, seed dispersal, seed dispersal syndrome

## Abstract

Although many plants are dispersed by wind and seeds can travel long distances across unsuitable matrix areas, a large proportion relies on co‐evolved zoochorous seed dispersal to connect populations in isolated habitat islands. Particularly in agricultural landscapes, where remaining habitat patches are often very small and highly isolated, mobile linkers as zoochorous seed dispersers are critical for the population dynamics of numerous plant species. However, knowledge about the quali‐ or quantification of such mobile link processes, especially in agricultural landscapes, is still limited. In a controlled feeding experiment, we recorded the seed intake and germination success after complete digestion by the European brown hare (*Lepus europaeus)* and explored its mobile link potential as an endozoochoric seed disperser. Utilizing a suite of common, rare, and potentially invasive plant species, we disentangled the effects of seed morphological traits on germination success while controlling for phylogenetic relatedness. Further, we measured the landscape connectivity via hares in two contrasting agricultural landscapes (simple: few natural and semi‐natural structures, large fields; complex: high amount of natural and semi‐natural structures, small fields) using GPS‐based movement data. With 34,710 seeds of 44 plant species fed, one of 200 seeds (0.51%) with seedlings of 33 species germinated from feces. Germination after complete digestion was positively related to denser seeds with comparatively small surface area and a relatively slender and elongated shape, suggesting that, for hares, the most critical seed characteristics for successful endozoochorous seed dispersal minimize exposure of the seed to the stomach and the associated digestive system. Furthermore, we could show that a hare's retention time is long enough to interconnect different habitats, especially grasslands and fields. Thus, besides other seed dispersal mechanisms, this most likely allows hares to act as effective mobile linkers contributing to ecosystem stability in times of agricultural intensification, not only in complex but also in simple landscapes.

## Graphical abstract

The most critical seed characteristics for successful endozoochorous seed dispersal by hares minimize exposure of the seed to the digestive system (i.e., dense seeds with a small surface area and an elongated shape). In addition, a hare's retention time is long enough to interconnect different habitats, especially grasslands and crop fields. Thus, besides other seed dispersal mechanisms, this most likely allows hares to act as effective mobile linkers contributing to ecosystem stability during times of agricultural intensification.

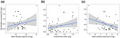

## 1 INTRODUCTION

In recent decades, we are witnessing a massive loss of biodiversity in flora and fauna (Cardinale et al., 2012; Chase et al., 2020; Pimm et al., 1995; Sala et al., 2000), with habitat loss and fragmentation as two major drivers (Estreguil et al., 2013; Rogan & Lacher, 2018; Tscharntke et al., 2005). Habitat fragmentation may lead to movement or dispersal barriers not only for animals (Andren, 1994; Crooks et al., 2011, 2017; Steffan‐Dewenter & Tscharntke, 1999) but also for plants (Estreguil et al., 2013; Malcolm et al., 2002; Rogan & Lacher, 2018; Tscharntke et al., 2005). Although many seeds are dispersed by wind and can travel long distances across unsuitable matrix areas, a large proportion relies on co‐evolved zoochorous seed dispersal (Cousens et al., 2010) to connect populations from isolated habitat islands. The animals carrying seeds to distant habitats function as mobile linkers (Jeltsch et al., 2013; Lundberg & Moberg, 2003) and are critical for the population dynamics of numerous plant species in various ecosystems (Jordano et al., 2007; Pakeman, 2001; Sasal & Morales, 2013), leading at best to a restoration of disturbed sites (Lundberg & Moberg, 2003). Especially in intensively used agricultural landscapes where remaining habitat islands are often very small and highly isolated, dependence on zoochorous seed dispersal makes plant species particularly vulnerable (Rogan & Lacher, 2018). Hence, seed dispersal has become a major constraint on establishing plant communities and restoring isolated habitat patches (Pywell et al., 2002).

In endozoochorous systems, seed dispersal success is often explained through the respective suite of seed traits i.e., *seed dispersal syndrome*, but see Green et al. (2021) that may influence ultimate germination after gut passage (Johnson et al., 2019; Pakeman et al., 2002). For example, comparatively small and light seeds were found to enhance dispersal success via sheep *Ovis gmelini aries*, rabbits *Oryctolagus cuniculus* (Pakeman et al., 2002), fallow deer *Dama dama* (Mouissie, Van der veen, et al., 2005), or waterfowl (Lovas‐Kiss et al., 2020; Soons et al., 2008). Also, denser seeds (mass/volume ratio) show increased dispersal rates when digested by cattle (Gardener et al., 1993; Simao Neto & Jones, 1987) and sheep (Russi et al., 1992). Further, seed shape e.g., rounder seeds, as measured by, e.g., the Flatness‐ or the Eccentricity index (Cervantes et al., 2016), were shown to positively influence germination success after dispersal by ungulates (Heinken et al., 2002; Mouissie, Van der veen, et al., 2005; Pakeman et al., 2002).

Recently, studies on the *dispersal syndrome* integrate phylogenetic relatedness of plant species (Boedeltje et al., 2015; D’hondt & Hoffmann, 2011; Lovas‐Kiss et al., 2020) and found that not only morphological seed traits but also the taxonomic relatedness of plant species needs to be considered when determining seed survival rates after complete digestion. In general, the species are seen as non‐independent since phylogenetically close species tend to be similar (Grafen, 1989). The closer two plant species are phylogenetically, the more similar their seed composition is, therefore, their resistance to the digestive system of the animals (Burns & Strauss, 2012). Moreover, since closely related species share similar traits, it is unclear whether phylogenetic relatedness promotes the patterns attributed to a particular trait or whether there is a causal relationship with the trait per se (Lovas‐Kiss et al., 2020).

While current research mainly focuses on long‐range epi‐ and endozoochorous seed dispersal by large herbivores (especially ungulates, see Albert et al., 2015; Baltzinger et al., 2019), smaller mammals seem to be rather understudied despite their dispersal potential (e.g., Fischer & Türke, 2016; Lessa et al., 2019; Naoe et al., 2019). For example, the European brown hare (*Lepus europaeus*, hereafter referred to as “hare”) is a typical medium‐sized herbivorous mammal (body mass 3.5–5 kg (Zachos, 2016)) in agricultural landscapes feeding on various wild herbs, grasses, and field crops (Schai‐Braun et al., 2013; Tapper & Barnes, 1986; Vaughan et al., 2003). Seeds are part of their natural diet and are eaten actively or passively during foraging (Sokos et al., 2015). Due to their spacious home ranges (in complex agricultural landscapes, e.g., 4‐day range size = 8 ± 7.8 ha, in simple agricultural landscapes: 23.9 ± 18.2 (Ullmann et al., 2020)), hares move across different habitat types, disperse seeds within their fecal pellets (Schai‐Braun et al., 2015; Tapper & Barnes, 1986), and can overcome plant dispersal barriers (Eycott et al., 2007; Reitz et al., 1994; Schai‐Braun & Hackländer, 2014). Indeed, feces samples of European hares from different ecosystems contain various germinable seeds of many plant species, indicating their capacity as effective mobile linkers (Mediterranean: Izhaki & Ne’eman, 1997; Forest habitats: Heinken et al., 2001, Panter & Dolman, 2012; Mountainous landscapes: Henríquez et al., 2014).

Our study aims to disentangle the effects of the most common morphological seed traits (i.e., seed mass, seed density, seed shape) as well as seed surface area and its ratio to mass on germination success while controlling for phylogenetic relatedness. We present the first controlled feeding experiment with hares in which both the ratio of seed intake and the germination success after digestion were recorded. Including common, rare, and invasive plant species as well as common field crops, we selected 44 plant species of open landscapes fed to captive hares. We hypothesize that taking the phylogenetic relatedness into account, high density and a comparatively small surface are advantageous for the seed survival after digestion by hares.

Further, we assessed the potential of hares as mobile linkers (i.e., seed dispersers), measured by the connectedness of distinct habitat types through hares in two contrasting agricultural landscapes (simple landscapes with large field sizes vs. complex landscapes with comparatively small field sizes), using GPS‐based movement data. We expect that as field size increases, the potential for hares as mobile linker decreases, that is, hares connect fewer habitats in simple compared to complex landscapes.

## 2 METHODS

### 2.1 Seed characteristics

For our feeding experiment, we selected 44 arable plant species with different morphological seed traits. Seed traits (length^a^, width^a^, height^a^, volume^a^, mass^b^) were available on the CC‐BY^a^ (Ganhão & Dias, 2019, 38 of 44 species) database, the SID^b^ (SID Database, 2021, 43 of 44 species) or the LEDA^a,b^ (Kleyer et al., 2008, mass: 1 of 44 species, volume: 6 of 44 species) database (Table S3). For species with multiple entries, we calculated the respective mean values. No data regarding seed height were available for 6 species (14%); therefore, we completed the dataset through supplementary searches in different gray literature sources following Picard et al. (2016). To qualify seed morphology, we calculated the eccentricity index ($\text{EI}=\frac{\text{length}}{\text{width}}$) and the flatness index ($\text{FI}=\frac{\text{length}\;+\;\text{width}}{2\;∙\text{height}}$) following Cervantes et al. (2016), as well as seed density, that is, mass per volume. Additionally, we calculated shape type (variance in dimensions: $V_{s}=\sum \frac{{{(x}_{i}‐\;\bar{x})}^{2}}{3},\;\;\text{with}\;x_{1}=\frac{\text{length}}{\text{length}},\;x_{2}=\;\frac{\text{width}}{\text{length}},\;x_{1}=\;\frac{\text{height}}{\text{length}}$) following Bekker et al. (1998) to establish a proxy for seed surface area. For rather spheric seeds (*V_s_* < 1), we used the formula for elliptical and for rather slender elongated seeds (*V_s_* > 1) the formula for cylindrical objects, as well as the seed surface area‐to‐seed mass ratio.

### 2.2 Hare feeding experiment

We assessed the endozoochorous seed dispersal potential in a controlled feeding experiment with hares. Therefore, we tested the influence of seed morphological traits while considering phylogenetic relatedness on the germination success of the 44 plant species after intestinal passage. Germination rates were assessed twice before the feeding experiment to determine the seeds’ germination capacity and once to assess seedling survival after being digested by hares.

In two consecutive years, during May–July 2019 & 2020, a defined number of seeds (depending on availability: 685–1500, Table 1, Table S3) were fed to captive hares in Niederfinow (Brandenburg), at the field station of the Leibniz Institute for Zoo and Wildlife Research (IZW), Berlin, around 40 km north of Berlin. During the feeding experiment, hares were housed in 2‐m^2^ cages. The floors of the cages consisted of a plastic grid with a mesh size large enough for the fecal pellets to fall through and be collected from a wooden collector mounted underneath, but small enough to allow comfortable sitting and walking. The upper part of the cages was closed with a metal mesh and a roof, whereas the lower part with the collector was covered with a cotton cloth to avoid contamination of the samples with anemochoric dispersed diaspores. Food and water were offered ad libitum. Prior to the experiment, we carefully cleaned the cages and the fecal collector, covering the latter with thick paper to obtain a clean surface.

**TABLE 1:** Plant species and their corresponding seed characteristics

Genus	Species	Total seeds fed^a^	Germination temperature [°C]^b^	Germination capacity [%]	Standardized germination success [%]^c^	FI	EI	Mass [mg]	Volume [mm^3^]	Density [mg/mm^3^]	Area [mm^2^]^d^	Area/mass [mm^2^/mg]	Seed origin^e^	Neophyte^f^	Conservation status^g^
*Achillea*	*millefolium*	700	15/25	87	4.76	2.12	2.67	0.0002	0.3805	0.0005	5.6116	28058	TK	No	–
*Anthriscus*	*sylvestris*	685	15/25	0	–	7.11	6.17	0.0037	2.7061	0.0014	23.7055	6372	RH	No	–
*Armeria*	*maritima*	800	15/25	7	0.00	2.48	1.99	0.0014	0.6603	0.0021	7.9176	5655	RH	No	V
*Arnoseris*	*minima*	800	5/15	81	0.77	1.95	2.15	0.0004	1.7114	0.0002	5.2934	13966	SC	No	2
*Artemisia*	*vulgaris*	700	15/25	3	4.76	1.87	2.58	0.0002	0.3565	0.0004	4.4369	27730	TK	No	–
*Berteroa*	*incana*	700	15/25	38	0.00	1.83	1.18	0.0005	0.8440	0.0006	6.3010	12911	TK	Yes	–
*Brassica*	*napus*	800	15/25	99	0.13	3.70	1.13	0.0033	1.6999	0.0019	10.5926	3209	PP	Yes	–
*Bupleurum*	*rotundifolium*	800	5/15	84	0.00	3.59	2.15	0.0025	1.9856	0.0012	14.9075	6011	SC	No	2
*Capsella*	*bursa‐pastoris*	700	15/25	13	2.20	1.08	1.85	0.0001	0.0902	0.0011	1.6695	16695	TK	No	–
*Crepis*	*capillaris*	685	15/25	65	0.22	1.93	3.52	0.0002	0.1633	0.0015	3.3544	13976	RH	No	–
*Dactylis*	*glomerata*	800	15/25	75	0.33	5.27	4.77	0.0008	1.9152	0.0004	17.3540	21692	RH	No	–
*Daucus*	*carota*	800	15/25	88	0.14	3.70	1.85	0.0012	2.2454	0.0005	14.1852	11890	RH	No	–
*Elymus*	*repens*	685	15/25	35	3.34	8.07	4.52	0.0037	4.5846	0.0008	40.2640	10754	RH	No	–
*Epilobium*	*hirsutum*	685	15/25	80	0.73	0.93	2.31	0.0001	0.0370	0.0027	1.2335	12335	RH	No	–
*Festuca*	*rubra*	800	15/25	88	0.14	6.77	5.85	0.0012	2.2288	0.0005	24.1146	20095	RH	No	–
*Legousia*	*speculumveneris*	800	5/15	97	0.00	1.11	1.33	0.0002	0.1727	0.0010	2.3562	14058	SC	No	–
*Lithospermum*	*arvense*	800	5/15	96	0.13	5.19	1.58	0.0058	3.7585	0.0015	22.0733	3793	SC	No	V
*Lolium*	*perenne*	1500	15/25	87	0.92	6.82	4.32	0.0020	4.1263	0.0005	31.7648	15882	RH, TK	No	–
*Lotus*	*corniculatus*	800	15/25	74	0.51	1.90	1.20	0.0009	0.4686	0.0019	4.0355	4623	RH	No	–
*Lupinus*	*polyphyllus*	800	15/25	52	1.44	9.21	1.33	0.0212	25.5254	0.0008	58.9049	2778	SV	Yes	–
*Lythrum*	*salicaria*	700	15/25	9	9.52	0.43	2.00	0.0001	0.0048	0.0147	0.3142	4487	TK	No	–
*Malva*	*sylvestris*	800	15/25	93	0.13	3.38	1.17	0.0055	11.3008	0.0005	12.3484	2245	RH	No	–
*Matricaria*	*chamomilla*	700	15/25	62	2.76	3.19	4.39	0.0001	0.4556	0.0002	7.6111	84568	TK	No	–
*Matricaria*	*discoidea*	700	15/25	80	0.18	1.22	2.66	0.0001	0.3247	0.0004	1.8081	12823	TK	Yes	–
*Medicago*	*sativa*	800	15/25	95	0.53	3.94	1.72	0.0024	4.3937	0.0005	16.8454	7159	RH	Yes	–
*Neslia*	*paniculata*	800	15/25	7	0.00	3.54	1.26	0.0026	9.7147	0.0003	11.4291	4395	SC	No	3
*Oenothera*	*biennis*	700	15/25	77	1.11	2.14	1.75	0.0004	0.5551	0.0007	4.7397	11849	TK	Yes	–
*Onobrychis*	*viciifolia*	800	15/25	52	0.00	8.26	1.65	0.0178	13.1685	0.0014	39.3092	2208	RH	Yes	–
*Papaver*	*argemone*	800	5/15	71	1.76	0.96	1.90	0.0002	0.0546	0.0027	1.2504	8336	SC	No	–
*Poa*	*annua*	685	15/25	1	218.98	2.99	3.48	0.0003	0.5983	0.0005	7.8270	26089	RH	No	–
*Poa*	*trivialis*	700	15/25	33	4.76	2.78	4.46	0.0001	0.3283	0.0003	5.4201	54200	TK	No	–
*Scabiosa*	*columbaria*	800	15/25	11	0.00	7.55	3.05	0.0021	34.1292	0.0001	41.2797	19617	RH	No	–
*Silene*	*Latifolia, subsp. alba*	700	15/25	97	0.29	2.03	1.26	0.0009	0.8296	0.0011	5.7118	6222	TK	No	–
*Sorghum*	*bicolor*	800	15/25	100	0.25	9.60	1.31	0.0132	12.1953	0.0011	45.7504	3465	AS	Yes	–
*Stellaria*	*media*	700	15/25	27	1.06	1.43	1.11	0.0004	0.3814	0.0010	3.7335	9333	TK	No	–
*Taraxacum*	*officinale*	800	15/25	69	0.00	4.00	3.86	0.0007	1.4092	0.0005	12.6786	18112	RH	No	–
*Teesdalia*	*nudicaulis*	800	5/15	93	0.13	1.58	1.56	0.0003	0.6136	0.0005	3.9584	13509	SC	No	–
*Trifolium*	*hybridum*	800	15/25	99	0.13	1.62	1.16	0.0007	0.4587	0.0015	4.0134	5733	RH	Yes	–
*Trifolium*	*pratense*	1500	15/25	74.5	0.54	4.11	1.38	0.0013	4.6064	0.0003	21.0970	16228	RH, TK	No	–
*Trifolium*	*repens*	800	15/25	89	0.70	1.99	1.21	0.0007	0.9920	0.0007	6.2471	8924	RH	No	–
*Tripleurospermum*	*inodorum*	685	15/25	30	0.00	2.51	2.55	0.0004	2.5916	0.0001	7.1673	19153	RH	No	–
*Valerianella*	*dentata*	800	5/15	83	0.15	2.13	1.84	0.0009	0.6676	0.0013	5.2229	5803	SC	No	V
*Valerianella*	*rimosa*	800	5/15	68	2.57	3.54	1.00	0.0012	3.0434	0.0004	12.5664	10385	SC	No	3
*Viola*	*arvensis*	700	15/25	46	0.00	2.01	1.75	0.0006	0.4877	0.0012	4.7892	8402	TK	No	–

Note

Morphological seed traits were obtained from the SID (SID Database, 2021), LEDA (Kleyer et al., 2008), and CC‐BY Database (Ganhão & Dias, 2019). The seed trait "height" to calculate area and FI was additionally obtained through supplementary searches in different gray literature sources for *M*. *chamomilla*, *M*. *discoidea*, *N*. *paniculata*, *Scabiosa columbaria*, and *Tripleurospermum inodorum*.

^a^ Number of seeds overall fed, numbers <700 result from limited availability of the respective seeds.

^b^ Seeds were sown below or above ground depending on their preferences with a day/night cycle of 12 h/12 h.

^c^ Calculated in relation to “Germination capacity [%]” as $\frac{100\;\cdot \;\text{germinated seeds}\;(\text{feces})}{\left(\text{germinated seeds}\;\left(\text{control group}\right)\cdot \;\frac{\text{seeds fed}}{100}\right)}$.

^d^ Seed surface area was calculated with the formula for elliptical objects for seeds with (*V_s_* < 1), and cylindrical objects for seeds with (*V_s_* > 1). Variance in dimensions was calculated as: $V_{s}=\;\sum \frac{{{(x}_{i}‐\;\bar{x})}^{2}}{3},\;\;\text{with}\;x_{1}=\frac{\text{length}}{\text{length}},\;x_{2}=\frac{\text{width}}{\text{length}},\;x_{1}=\frac{\text{height}}{\text{length}}$ formula from Bekker et al. (1998).

^e^ Seeds were ordered at RH (Rieger‐Hofmann, https://www.rieger‐hofmann.de), TK (Templiner Kräutergarten, https://templiner‐kraeutergarten.de), AS (Asklepios Seeds, https://www.asklepios‐seeds.de), PP (Pflanzen‐ Pflanzen, https://www.pflanzen‐pflanzen.de), or SC (self‐collected in the field, Bavaria, Germany).

^f^ Status as a neophyte in German, data from: https://www.floraweb.de/, retrieved: 22.07.2021.

^g^ Conservation status in Germany (red list), data from: https://www.floraweb.de/, retrieved: 22.07.2021.

The feeding experiment was performed as an incomplete randomized block design due to the availability of hare individuals (2019: *n* = 8, 2020: *n* = 7; one hare was omitted because it refused to ingest the seeds). All hares received the same number of seeds of all plant species, but seed feeding was blocked in time, and not all plant species were offered simultaneously to a particular hare. Instead, different species combinations were fed to ease seedling identification by mixing them with regular hare food (nutritious pellets & oats). Seeds of respective 1 to 4 species (n per species = 100) were fed with a minimum in‐between break of 4 days to ensure all seeds were being entirely excreted or digested (retention time: 7 ± 1.4 h; Stott, 2008). During the following three days, feces were carefully collected daily and air‐dried in closed paper bags. Then, feeding was repeated 8 times, with each hare receiving different combinations until all seeds were fed (Table 1, Table S3).

### 2.3 Germination capacity of offered seeds

The germination capacity of the seeds was determined in a control group germination test. A priori, the seed samples were stratified at 4°C for 6 weeks to break seed dormancy. 100 seeds of each species were counted and, following Heinken et al. (2001), placed in plastic boxes (180 × 133 × 87 mm), pre‐filled with a 2‐cm layer of Seramis^®^ (clay granules) substrate, and 1 cm of germination soil. Boxes were covered with perforated lids and put into RUMED^®^ and Fitotron^®^ light cabinets for six weeks. The cabinets were set to a day/night rhythm of 12 h each, including 12 h of maximum lighting of 100%. Species were separated into two groups according to their preferred germination temperatures (SID Database, 2021: 5°C/15°C and 15°C/25°C, day/night respectively, Table 1). The humidity was visually controlled in a daily manner, and germination progress was recorded every 3 days. We used the germination capacity (control) results to calculate the standardized germination success of each plant species after gut passage $\left(\text{Standardized germination success}\;\left[\%\right]=\;\frac{100\;\cdot \;\text{germinated seeds}\;\left(\text{feces}\right)}{\left(\text{germinated seeds}\;\left(\text{control group}\right)\;\cdot \;\frac{\text{seeds fed}}{100}\right)}\right)$. Dry fecal samples were stratified in the same way as the seed samples in the control group. Prior to germination, pellets were soaked in distilled water and carefully opened with rounded glass sticks; then, they were planted analogously to the control group. Germination was recorded every three days, and seedlings were marked and identified as soon as they showed distinct characteristics.

### 2.4 Phylogeny

We extracted genomic DNA from our 44 different plant species (Table 1) from 100 to 150 mg of fresh plant tissue with a modified CTAB plant DNA extraction protocol following Inglis et al. (2018). During the elution process, the amount of TE buffer was reduced to 60 µl to ensure a sufficient final concentration of DNA. After a quantity check of the concentration and pureness via a spectral photometer (NanoDrop ND‐1000, Thermo Scientific^®^), the DNA was stored at −20°C.

We amplified a 633‐bp fragment of the nuclear ribosomal internal transcribed spacer 1 (ITS1) 5.8S and ITS2 to assess the species level and phylogenetic relationship. Polymerase chain reactions (PCRs) were performed according to previously published protocols (Cheng et al., 2016; White et al., 1990). We cleaned the amplified products using an ExoAP procedure followed by a sequencing reaction and Sanger sequencing (Applied Biosystems™ 3500 Genetic Analyzer). The ITS1–ITS2 region sequences were aligned with ClustalW (Larkin et al., 2007; Thompson et al., 2003) as implemented in Geneious v8.1.9 (Kearse et al., 2012).

**FIGURE 1:**
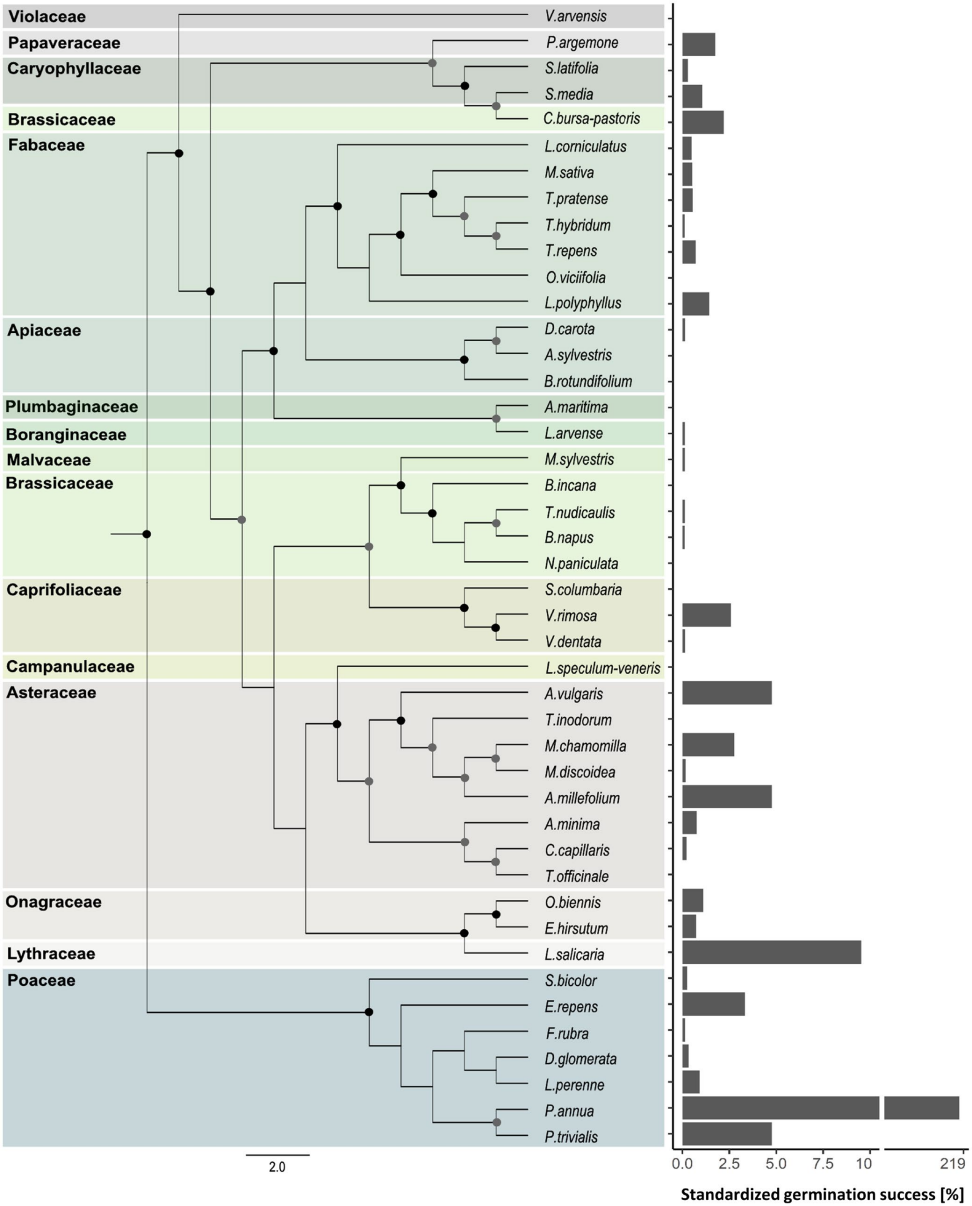
Left: phylogenetic maximum‐likelihood tree based on full ITS1–ITS2 region (including ITS1, 5.8S, and ITS2) sequences of 44 selected plants species. Dots indicate bootstrap values ≥65, while grey dots indicate bootstrap support of 65%–95%, and black dots indicate bootstrap support of 95%–100% of the branch splits. Right: Standardized germination success of seeds [%] after being digested by hares. *X*‐axis is split at 10% to obtain a better perspective

Phylogenetic trees were generated of 35 amplified ITS1–ITS2 region sequences and nine additional ITS1–ITS2 region sequences from GenBank (Clark et al., 2016) as the respective species had not grown sufficiently to extract enough DNA (*Anthriscus sylvestris*, *Crepis capillaris*, *Taraxacum officinale*, *Tripleurospermum inodorum*, *Artemisia vulgaris*, *Onobrychis viciifolia*, *Lythrum salicaria*, *Armeria maritima*, *Poa annua*; GenBank: GQ379320.1, AJ633381.1, AJ633290.1, JF907423.1, AM398927.1, AB854512.1, AY035750.1, AJ225574.1, GQ324485.1). The phylogenetic relationship was established with RAxML (version 8.0.0, Stamatakis, 2014) using the maximum‐likelihood algorithm as well as the GTRGAMMAI substitution model (Yang, 1993) with 10,000 bootstrap replicates. The resulting best tree (Figure 1) given by RAxML was used in all downstream analyses.

### 2.5 Hares as mobile linkers in contrasting agricultural landscapes

To investigate the potential of seed dispersal by hares throughout the landscape, we explored the connectedness of distinct habitat types (grassland, forest, field, wetland, quarry, and urban) with movement data of 63 GPS‐collared hares in two contrasting landscapes. One study site represented a simple landscape in northeast Germany, Brandenburg, about 100 km north of Berlin with an average field size of 27.5 ± 1.1 ha, covered up to 62% by arable land (study site hereafter referred to as "simple landscape"). The second study site was located in South Germany, Bavaria, about 50km north of Munich, with comparatively small field sizes of 2.9 ± 0.04 ha, covered by 66% of arable lands (study site hereafter referred to as "complex landscape").

We created a dataset using GPS data of 48 hares from Ullmann et al. (2018), Ullmann et al. (2020), caught in 2014 and 2015 (27 in the simple landscape, 21 in the complex landscape) combined with additional 15 individuals caught during 2019 and 2020 in the simple landscape. The 63 hares were caught, handled according to Ullmann et al. (2018), and the corresponding data were stored in the Movebank data repository (Wikelski et al., 2020). All individuals used for our analysis were tracked for a minimum of 10 days. The GPS resolution was adjusted to hourly GPS fixes, resulting in a total of 62,528 GPS locations. For further description of the study sites, GPS collaring, and data storage, see Ullmann et al. (2018) and Mayer et al. (2018). Land‐use types and GPS data were used from published data (Mayer et al., 2018; Ullmann et al., 2018, 2020). Animal tracking was obtained in accordance with the Federal Nature Conservation Act (§ 45 Abs. 7 Nr. 3) and approved by the local nature conservation authority (reference numbers: 2347‐6‐2019, LUGV V3‐ 2347‐22‐2013, and 55.2‐1‐54‐2532‐229‐13).

Generally, we were interested in the potential of hares to connect habitats of differing or similar land‐use types in the two contrasting landscapes. Hence, we first calculated distances between all habitat patches with at least one GPS location as follows. We used QGIS (QGIS Development Team, 2021) to calculate the centroids of all visited habitat patches and derived a distance matrix using the R packages sf (Pebesma, 2018) and rgeos (Bivand & Rundel, 2020). Then, we compared the centroid distances of differing land‐use types and similar land‐use types to the mean Euclidean distances hares traveled within their retention time interval of 7 ± 1.4 h (Stott, 2008) between the two contrasting landscapes. More specifically, we performed the analysis for retention times of mean − CI: 5.6 h, mean: 7 h, and mean + CI: 8.4 h, respectively, and report the overall mean values. The distances were estimated using the R package amt (Signer et al., 2019).

Particularly, we were interested in the amount of realized connections of patches through hares. Therefore, we calculated how many differing land‐use types and unique patches of the same land‐use type each hare visited on average during the retention time intervals. For this purpose, we first assigned the land‐use type (grassland, forest, crop field, wetland, quarry, and urban) and the unique patch id to all GPS locations. Second, we generated initial tracks of consecutive GPS locations lasting 5.6, 7, and 8.4 h for each individual and counted the number of land‐use types and single, unique patches of the same land‐use type visited. Third, we shifted these tracks to subsequent GPS locations and repeated counting until the end of the observation time (moving window). Finally, we averaged the number of visited land‐use types for each retention time interval and unique patches per land‐use type for each individual and compared those between the two different landscapes.

### 2.6 Statistical analysis

All analyses were conducted in R version 4.0.2 (R Core Team, 2020), R Studio version 1.2.5019 (R Studio Team, 2019), and QGIS (QGIS Development Team, 2021). We used the R package dplyr (Wickham et al., 2021) for data management and ggplot2 (Wickham, 2016) for figure generation.

### 2.7 Germination success

A priori, we excluded *Anthriscus sylvestris* from further analysis as it did not germinate in the control group. Subsequently, we excluded seed traits that correlated with each other (flatness index, mass, volume, surface/mass ratio; Pearson correlation coefficient >0.7, Figure S1) from further analysis (Dormann et al., 2013) and performed a PCA to select the variables for subsequent analysis, which explained most of the variance (Figure S2). We used a general linear mixed model to investigate how standardized germination success was related to seed traits (seed density, Eccentricity index, and seed surface area). Markov chain Monte Carlo (MCMC) was implemented to estimate the influence of predictor on response variables using the R package MCMCglmm (Hadfield, 2010) with germination rate as the dependent variable and the seed traits included as fixed effects (*n* iterations = 5,000,000, burn‐in = 50,000, thin = 500). To identify the model that explained most of the variance, we performed model selection (Appendix: Table S1) based on the deviance information criterion (DIC) using the dredge function implemented in the R package MuMIn (Barton, 2016). Following the studies of Burnham and Anderson (1998), we used the model with the highest DIC score (lowest DIC value) to explain our data. All models within 2 DIC units were considered as competing models (Spiegelhalter et al., 2002).

We repeated the analysis using Bayesian MCMCglmm, including phylogenetic inertia (i.e., a measure of branch length from each species) as a random effect in the model (*n* iterations = 5,000,000, burn‐in = 50,000, thin = 500). We used the same dependent and explanatory variables combined with a correlation structure based on the phylogenetic tree (Figure 1) for subsequent model selection (Appendix: Table S2). To evaluate whether the inclusion of the phylogenetic data improved our model, we compared their DIC values. Following others, we calculated Lynch's phylogenetic heritability as a phylogenetic signal measure and report the posterior mean heritability and the 95% interval of highest posterior density (HPD) based on MCMC draws from the marginal posterior distribution (Lovas‐Kiss et al., 2020). The response variable was log (*x* + 1)‐transformed (to include zeros) for both models to obtain a distribution approximate to normal (Mangiafico, 2017); predictor variables were log‐transformed to reduce heteroscedasticity.

## 3 RESULTS

Out of 34,710 seeds from 44 plant species fed, 177 seedlings of 33 species emerged from feces (0.51%). Considering the species‐specific germination capacity, the standardized germination success was 6% in total. Standardized germination success varied largely among species (max: 219% for *P*. *annua*) and was higher for non‐neophytes (mean: 1.27%) than for neophytes (mean: 0.42%) and for endangered species (mean: 1.21%) compared with non‐endangered ones (mean: 0.52%) without *P*. *annua* into account (Table 1).

### 3.1 Phylogenetic reconstruction

The maximum‐likelihood tree calculated from a 633‐bp‐long fragment containing ITS1, 5.8S and ITS2 shows a well‐separated phylogeny of the 15 different families, supported by generally fairly high bootstrap values (65%‐95% indicated by gray dots, >95%‐100% indicated by black dots; Figure 1). The division between monocotyledons (here grasses) and dicotyledons was confirmed with a bootstrap value of 100%. Furthermore, within the dicotyledons, the maximum‐likelihood tree reflects a clear separation according to the family's origin, supporting the robustness of the data analysis.

### 3.2 Seed traits and their influence on germination success

**FIGURE 2:**
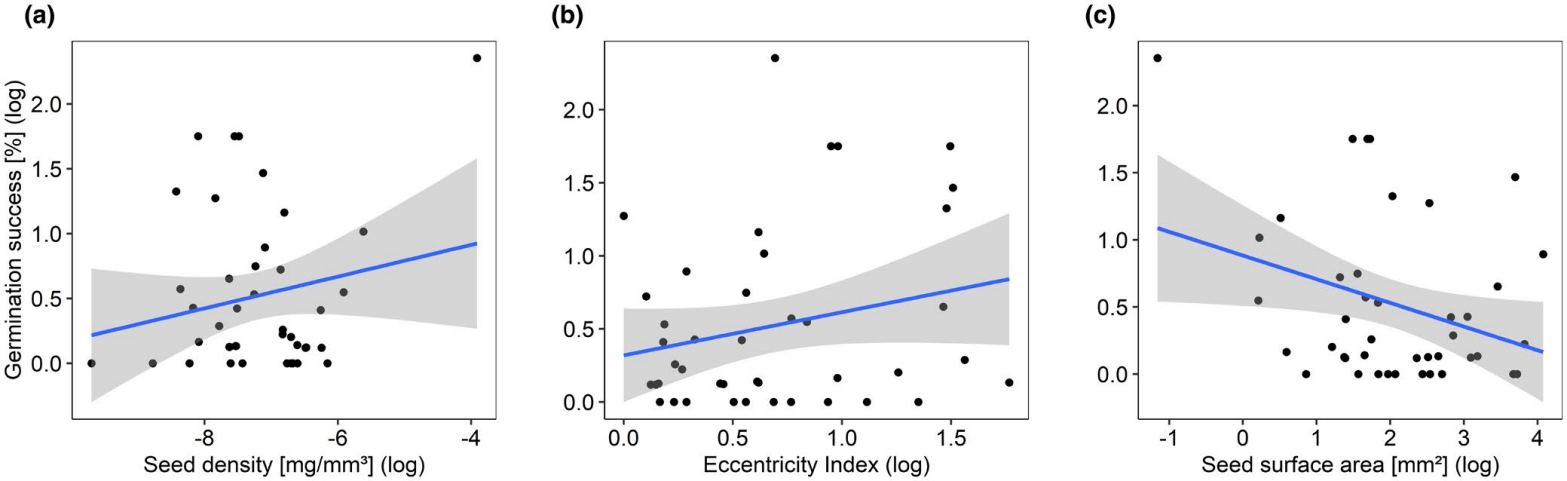
Dependence of standardized germination success on (a) seed density, (b) seed shape as measured by the Eccentricity index and seed surface area (*n* = 43). Observed values (circles), predicted values (blue line), and confidence intervals (gray shading) for the MCMC‐GLMMs. Graphs are shown without the outlier *P*. *annua* to obtain a better perspective

Seed's germination was linked to the three covariates: seed density, Eccentricity index (EI), and seed surface area (Figure 2), as shown by all competing models within 2 DIC units. Rather long, elongated seeds (increasing EI), an increasing seed density, and a decreasing seed surface area were positively related to standardized germination success after gut passage. Using an MCMCglmm with phylogeny as a random effect significantly increased model convergence compared with the models without phylogeny (best‐fit models: Δ DIC =3.8, Tables S1 and S2).

Heritability as measured by Lynch's signal (mean = 0.046, 95%, HPD interval 0.0014–0.14) significantly influenced the model outcome.

### 3.3 Mobile link potential of hares in agricultural landscapes

**FIGURE 3:**
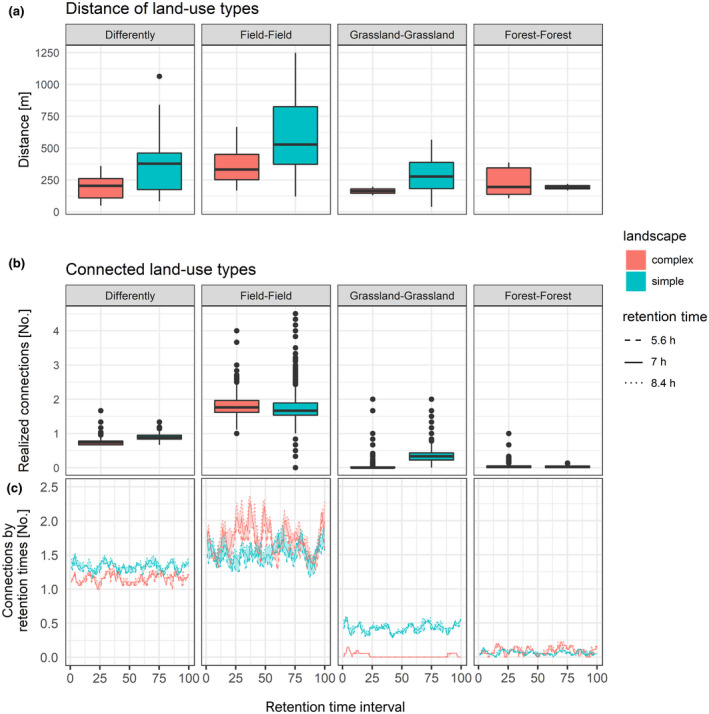
(a) Average distances between land‐use types in complex and simple landscapes. (b) Average number of connected land‐use types of hares within the retention time of 7 ± 1.4 h (moving window approach) in complex and simple landscapes. (c) Number of connected land‐use types of hares within the retention time intervals of 5.6, 7, and 8.4 h (moving window approach) in complex and simple landscapes. Left to right: Differing habitat types include distances (a), realized connections (b), and connections by retention time (c) between grassland, crop field, wetland, forest, quarry, or urban; between fields; grasslands; and forest patches. Land use types were recorded from 62,528 GPS locations of 63 individual hares (42 in simple, 21 in complex landscapes)

The distance to differing land‐use types (simple: 376 ± 241 m; complex: 192 ± 97 m), the distance from crop field to crop field (simple: 579 ± 302 m; complex: 365 ± 149 m), and the distance from grassland to grassland (simple: 289 ± 151 m; complex: 163 ± 47 m) were larger in simple versus complex landscapes, while that from forest to forest (simple: 193 ± 36 m; complex: 232 ± 125 m) was smaller (Figure 3a). Hares in simple landscapes moved 1.30 ± 0.28 km per ~7 ± 1.4 h retention time interval (i.e., potential dispersal distance), while those in complex landscapes traveled 0.77 ± 0.17 km during the same period.

Comparing both study sites, hares connected on average slightly more differing land‐use types in simple landscapes (1.30 ± 0.16 m) than in complex landscapes (mean: 1.10 ± 0.15) within a 7 ± 1.4 h period. Hares in complex landscapes connected more fields, fewer isolated grassland patches, and a similar amount of forest than in simple landscapes (Figure 3b,c).

Across both regions, hares connected mainly various crop fields (70.78 ± 1.96%). To a lesser extent, they connected crop fields with grasslands (12.72 ± 1.25%), grasslands with grasslands (4.34 ± 0.53%), and crop fields with forests (2.95 ± 0.55%).

## 4 DISCUSSION

### 4.1 Seed characteristics

With this controlled feeding experiment, we provide fundamental advances in understanding the potential impacts of mobile linkers as seed dispersers. Hitherto our understanding of the efficiency of smaller mammals and their potential impacts on plant communities and recruitment was limited, as most of the literature focuses on rather large mammals with more extensive home ranges (Albert, Mårell, et al., 2015, Karimi et al., 2020, Mouissie, Vos, et al., 2005, but see, e.g., Lessa et al., 2019, Naoe et al., 2019, Yang et al., 2019). Our feeding experiment demonstrates that 32 out of 42 species that germinated in the control group also survived the gut passage of hares and germinated afterward. However, with one exception (*P*. *annua*), all species showed lower germination rates after gut passage in relation to the control group. Indicated by the range of germination rates and similar to findings from Milotić and Hoffmann (2016), germination success was clearly taxon‐dependent. To understand the mechanisms behind successful germination, most endozoochoric studies consider seed traits and their influence on dispersal rates exclusively (e.g., Cosyns et al., 2005; Mouissie, Vos, et al., 2005; Pakeman et al., 2002). According to our study, germination success depends both on such morphological traits and on taxon‐specific additional factors, such that neglecting phylogenetic affinity of the observed species may compromise our understanding of endozoochoric seed dispersal—and ultimately germination success—by mobile linkers (Boedeltje et al., 2015; D’hondt & Hoffmann, 2011; Lovas‐Kiss et al., 2020).

Our best‐fit model revealed that consistent with our hypotheses, denser seeds with comparatively small surface areas are positively related to germination success. Contrary to our expectations, not rounder but rather long, elongated seeds show higher germination rates after being digested by hares.

Consistent with our findings, increasing seed hardness was identified as the most critical factor for gut passage survival in mallards (*Anas platyrhynchos*) in a study of waterbirds (Lovas‐Kiss et al., 2020) after controlling for phylogeny. Kleyheeg et al. (2018), Kleyheeg et al. (2018) demonstrated that harder seeds are more likely to survive the gizzard of mallards, where mechanical digestion occurs before the seeds are further released into the intestine. Therefore, harder seeds are more likely to survive intestinal passage and are egested over a more extended range of time, increasing the maximum putative dispersal distance (Farmer et al., 2017; Kleyheeg et al., 2019). Although the digestive systems of birds and mammals are very different, it stands to reason that specific characteristics of seeds determine their survival after digestion irrespective of the respective mobile linker. The importance of such seed traits seems evident as, for example, birds and ungulates often disperse the same plant species. (Albert, Auffret, et al., 2015; Lovas‐Kiss et al., 2019, 2020; Soons et al., 2008).

Furthermore, we found a decreasing seed surface area as a significant driver for successful germination. Thus, we assume that dense and heavy seeds with relatively small surface areas enhance seed dispersal via hares as such seeds seem to be better protected from the milieu prevailing in the stomach. Hence, we conclude that most seeds will lose their germination capacity inside the digestive system. This is consistent with the findings that dense and small seeds are superior in endozoochoric seed dispersal by mammals (Albert, Auffret, et al., 2015; Albert, Mårell, et al., 2015; Bourgeois et al., 2005; D’hondt & Hoffmann, 2011; Lepková et al., 2018; Shiels & Drake, 2011; Williams et al., 2000) or waterbirds (Lovas‐Kiss et al., 2020). We did expect rounder, more spheric seeds to show higher germination rates as found, for example, as shown for ungulates (Heinken et al., 2002; Mouissie, Van der veen, et al., 2005; Pakeman et al., 2002). The finding that elongated seeds are superior in surviving digestion might be reasoned through the digestive system of hares. However, this is purely speculative. Besides, in a feeding experiment by Cosyns et al. (2005), more elongated seeds were also shown to be positively related to germination success after digestion by rabbits, cattle (*Bos taurus*), donkeys (*Equus asinus*), and horse (*Equus cabbalus*). Another explanation might be that for our selection of plants, the Eccentricity index was interdependent with the surface to mass ratio, and therefore, similar to seeds with less area, digestion processes have less contact area to break down the seeds. In summary, the most critical seed characteristics for successful endozoochorous seed dispersal minimize exposure of the seed to the stomach/gut and their associated digestive fluids (i.e., dense seeds with less seed surface area). In addition, supported by the inclusion of phylogeny and that some seeds with similar traits show different germination rates, we argue that specific compositions of the seed coat are better adapted to survive digestion than others.

### 4.2 Hares as mobile linkers

Connecting fragmented habitat patches is essential for zoochorous plant species as it helps to stabilize biodiversity in fragmented landscapes (Damschen et al., 2019; Lundberg & Moberg, 2003), where many animal species provide effective functional connectivity and seed dispersal via endozoochory (Pellerin et al., 2016; Williams et al., 2008).

We could show that hares connect different habitats in simple and complex agricultural landscapes within their species‐specific retention time and, therefore, can act as mobile linkers. Despite the relatively low germination rates after hare's digestion, we emphasize that long‐distance dispersal through endozoochory might have disproportional large effects on plant community composition and species persistence (Nathan et al., 2008; Schurr et al., 2009). Considering the number of seeds produced per plant, ingested by individual hares throughout the year and subsequently transported to sites that may be more favorable for germination, the total amount may be quite significant. Contrary to our prediction, the number of interconnected habitats with different land‐use types was similar in both landscapes. Although hares moved twice the daily distance in simple compared to complex landscapes, they connected more crop fields with crop fields in the latter landscape, probably because field sizes were about 10% of those in simple landscapes. Surprisingly though, more grasslands were connected in simple landscapes. We argue that hares need to travel more often to such high‐quality foraging habitats. Both grasslands and field margins contain a higher plant species diversity than their arable surroundings (Marshall & Moonen, 2002; Rosado & de Mattos, 2017) but are simply less available in a landscape with comparatively large fields. A higher plant diversity, found in field margins and grasslands, is associated with health benefits for hares, and there is a substantial selection for a highly diverse diet (Reichlin et al., 2006). Moreover, the observed loss of high‐quality habitat patches with wild herbs may be related to decreasing hare populations (Hackländer, 2002). The non‐existent difference in forest patch connections seems justified, as the distance of such habitats is similar in both areas.

Conclusively, hares seem to be well adapted to both simple and complex landscapes and connect several habitats while foraging. Thus, hares as mobile linkers seem to play an important role in determining local plant communities (Lundberg & Moberg, 2003). These mobile linkers, especially on human‐disturbed land such as agricultural landscapes, might be critical acting as mediators of recolonization through seed import from off‐sites (i.e., grasslands or field margins) to patches where the resources for natural succession are impoverished (Duncan & Chapman, 1999; Lundberg & Moberg, 2003). In this sense, many mobile linkers are essential factors determining the direction of ecosystem development following a disturbance (Cox & Elmqvist, 2000; Nyström & Folke, 2001).

Our results suggest that management plans in agricultural landscapes should consider the functional role of mobile linkers in maintaining ecosystems and contributing to ecosystem resilience. This is even more evident as especially rare and non‐neophytic species achieved higher germination rates in our study. For the support of hares, this would imply maintaining a high plant diversity at the field margins, for example, through the establishment of flowering strips or the temporary set aside of fields.

## CONFLICT OF INTEREST

The authors declare no conflict of interest.

## AUTHOR CONTRIBUTIONS

**Jonas Stiegler:** Conceptualization (lead); Formal analysis (lead); Investigation (lead); Methodology (equal); Visualization (lead); Writing – original draft (lead). **Katrin Kiemel:** Conceptualization (supporting); Formal analysis (supporting); Methodology (supporting); Visualization (supporting); Writing – review & editing (supporting). **Jana Eccard:** Writing – review & editing (supporting). **Christina Fischer:** Writing – review & editing (supporting). **Robert Hering:** Conceptualization (supporting); Formal analysis (supporting); Methodology (supporting); Visualization (supporting); Writing – review & editing (supporting). **Sylvia Ortmann:** Writing – review & editing (supporting). **Lea Strigl:** Investigation (supporting); Methodology (supporting); Writing – review & editing (supporting). **Ralph Tiedemann:** Writing – review & editing (supporting). **Wiebke Ullmann:** Data curation (supporting); Writing – review & editing (supporting). **Niels Blaum:** Conceptualization (equal); Methodology (equal); Writing – review & editing (lead).

## Supporting information

Supplementary Material

## Data Availability

Data files to perform the analyses are supplied as Supporting Information at the Dryad data repository at https://doi.org/10.5061/dryad.98sf7m0k6. The sequence data used to build the phylogenetic tree are openly available on GenBank of NCBI at https://www.ncbi.nlm.nih.gov/ under the accession numbers GQ379320.1, AJ633381.1, AJ633290.1, JF907423.1, AM398927.1, AB854512.1, AY035750.1, AJ225574.1, GQ324485.1, and OL415903‐OL415937.
