# Supplementary material for: Seed traits matter—Endozoochoric dispersal through a pervasive mobile linker
Source: Ecol Evol. 2021 Dec 14;11(24):18477–91. doi: 10.1002/ece3.8440 (PMC8717309; doi:10.1002/ece3.8440)
Supplement: Supplementary file 1 — Supplementary Material [file ECE3-11-18477-s001.docx]

**Supporting information**

**Table S1: Model selection table without phylogeny, ranked by DIC^⸸^**

| *model* | *(Int)* | *log(density)* | *log(EI)* | *log(seed surface area)* | *df* | *logLink* | *DICc* | *delta* | *weight* |
| --- | --- | --- | --- | --- | --- | --- | --- | --- | --- |
|  | | | | | | | | | |
| 6 | 0.632 | 0.612 |  | -0.207 | 4.000 | -57.632 | 119.147 | 0.000 | 0.295 |
| 2 | 0.248 | 0.554 |  |  | 3.000 | -58.425 | 119.760 | 0.613 | 0.217 |
| 4 | 1.239 | 0.636 | 0.146 |  | 4.000 | -58.535 | 120.961 | 1.815 | 0.119 |
| 8 | 0.877 | 0.631 | 0.041 | -0.190 | 5.000 | -58.168 | 121.182 | 2.035 | 0.106 |
| 1 | 0.634 |  |  |  | 2.000 | -59.682 | 121.276 | 2.129 | 0.102 |
| 5 | 0.997 |  |  | -0.177 | 3.000 | -59.306 | 121.531 | 2.385 | 0.089 |
| 3 | 1.002 |  | 0.051 |  | 3.000 | -60.162 | 123.209 | 4.062 | 0.039 |
| 7 | 0.615 |  | -0.060 | -0.200 | 4.000 | -59.796 | 123.477 | 4.330 | 0.034 |

⸸ the initial model was calculated with the 3 covariates 'density', 'EI' and 'seed surface area' as $y\sim N\left( \mu, \sigma^{2}I \right) with \mu=\beta_{0}+ \beta_{1}x_{1}+ \beta_{2}x_{2}+\beta_{3}x_{3}$.

**Table S2: Model selection table including phylogeny, ranked by DIC^⸸^**

| *model* | *(Int)* | *log(density)* | *log(EI)* | *log(seed surface area)* | *df* | *logLink* | *DICc* | *delta* | *weight* |
| --- | --- | --- | --- | --- | --- | --- | --- | --- | --- |
|  | | | | | | | | | |
| 5 | 1.196 |  |  | -0.242 | 4.000 | -52.316 | 115.389 | 0.000 | 0.283 |
| 1 | 0.682 |  |  |  | 3.000 | -53.210 | 116.171 | 0.782 | 0.192 |
| 6 | 0.973 | 0.320 |  | -0.248 | 5.000 | -53.366 | 117.128 | 1.739 | 0.119 |
| 7 | 0.863 |  | -0.055 | -0.267 | 5.000 | -52.859 | 117.137 | 1.748 | 0.118 |
| 3 | 1.313 |  | 0.088 |  | 4.000 | -53.154 | 117.234 | 1.845 | 0.113 |
| 2 | 0.483 | 0.271 |  |  | 4.000 | -54.172 | 117.830 | 2.441 | 0.084 |
| 8 | 0.885 | 0.306 | -0.017 | -0.254 | 6.000 | -53.852 | 118.880 | 3.491 | 0.049 |
| 4 | 1.363 | 0.339 | 0.130 |  | 5.000 | -54.372 | 119.220 | 3.831 | 0.042 |

⸸ the initial model was calculated with the 3 covariates 'density', 'EI' and 'seed surface area' as $y\sim N\left( \mu+u, \sigma^{2}I \right) with \mu=\beta_{0}+ \beta_{1}x_{1}+ \beta_{2}x_{2}+\beta_{3}x_{3}and u$ = a random effect with a correlation matrix based on the phylogenetic tree.

| \| **Table S3: Plant species and their initially considered seed characteristics** \| \| --- \| | | | | | | | | | | | | | | | | | | |
| --- | --- | --- | --- | --- | --- | --- | --- | --- | --- | --- | --- | --- | --- | --- | --- | --- | --- | --- | --- |
| **Family** | **Genus** | **Species** | **Year ^α^** | **Seeds fed^β^** | **Germination control group** | **Germination feces** | **V_s_ ^µ^** | **Length**  **[mm]** | **Width**  **[mm]** | **Height**  **[mm]** | **Volume**  **[mm^3^]^µ^** | **Mass**  **[mg]** | **EI** | **FI** | **Density**  **[**$\frac{\boldsymbol{mg}}{\boldsymbol{mm}^{\boldsymbol{3}}}$**]** | **Area**  **[mm^2^]** | **Surface/mass** |  |
| Apiaceae | *Anthriscus* | *sylvestris* | 2020 | 685 | 0 | 0 | 1.376 | 6.562 | 1.063 | 1.022 | 2.706 | 0.0037 | 6.170 | 7.110 | 0.0014 | 23.706 | 6372 |  |
| Apiaceae | *Bupleurum* | *rotundifolium* | 2019 | 800 | 84 | 0 | 1.045 | 2.879 | 1.338 | 1.057 | 1.986 | 0.0025 | 2.152 | 3.586 | 0.0012 | 14.908 | 6011 |  |
| Apiaceae | *Daucus* | *carota* | 2019 | 800 | 88 | 1 | 0.972 | 2.894 | 1.560 | 1.034 | 2.245 | 0.0012 | 1.854 | 3.700 | 0.0005 | 14.185 | 11890 |  |
| Asteraceae | *Achillea* | *millefolium* | 2020 | 700 | 87 | 29 | 1.137 | 2.003 | 0.751 | 0.300 | 0.380 | 0.0002 | 2.668 | 2.116 | 0.0005 | 5.612 | 28058 |  |
| Asteraceae | *Arnoseris* | *minima* | 2019 | 800 | 81 | 5 | 1.045 | 1.717 | 0.797 | 0.587 | 1.711 | 0.0004 | 2.155 | 1.950 | 0.0002 | 5.293 | 13967 |  |
| Asteraceae | *Artemisia* | *vulgaris* | 2020 | 700 | 3 | 1 | 1.124 | 1.749 | 0.677 | 0.350 | 0.356 | 0.0002 | 2.584 | 1.867 | 0.0004 | 4.437 | 27731 |  |
| Asteraceae | *Crepis* | *capillaris* | 2020 | 685 | 65 | 1 | 1.235 | 1.814 | 0.515 | 0.450 | 0.163 | 0.0002 | 3.519 | 1.930 | 0.0015 | 3.354 | 13977 |  |
| Asteraceae | *Matricaria* | *chamomilla* | 2020 | 700 | 62 | 12 | 1.299 | 3.089 | 0.704 | 0.290 | 0.456 | 0.0001 | 4.388 | 3.191 | 0.0002 | 7.611 | 84568 |  |
| Asteraceae | *Matricaria* | *discoidea* | 2020 | 700 | 80 | 1 | 1.136 | 1.136 | 0.427 | 0.410 | 0.325 | 0.0001 | 2.661 | 1.223 | 0.0004 | 1.808 | 12823 |  |
| Asteraceae | *Taraxacum* | *officinale* | 2019 | 800 | 69 | 0 | 1.263 | 3.712 | 0.963 | 0.600 | 1.409 | 0.0007 | 3.856 | 4.000 | 0.0005 | 12.679 | 18112 |  |
| Asteraceae | *Tripleurospermum* | *inodorum* | 2020 | 685 | 30 | 0 | 1.118 | 2.205 | 0.865 | 0.710 | 2.592 | 0.0004 | 2.549 | 2.512 | 0.0001 | 7.167 | 19154 |  |
| Boranginaceae | *Lithospermum* | *arvense* | 2019 | 800 | 96 | 1 | 0.887 | 3.331 | 2.109 | 1.767 | 3.759 | 0.0058 | 1.579 | 5.194 | 0.0015 | 22.073 | 3794 |  |
| Brassicaceae | *Berteroa* | *incana* | 2020 | 700 | 38 | 0 | 0.713 | 1.538 | 1.304 | 0.440 | 0.844 | 0.0005 | 1.180 | 1.825 | 0.0006 | 6.301 | 12912 |  |
| Brassicaceae | *Brassica* | *napus* | 2019 | 800 | 99 | 1 | 0.687 | 1.954 | 1.726 | 2.027 | 1.700 | 0.0033 | 1.132 | 3.703 | 0.0019 | 10.593 | 3210 |  |
| Brassicaceae | *Capsella* | *bursa-pastoris* | 2020 | 700 | 13 | 2 | 0.972 | 0.993 | 0.535 | 0.336 | 0.090 | 0.0001 | 1.854 | 1.083 | 0.0011 | 1.670 | 16695 |  |
| Brassicaceae | *Neslia* | *paniculata* | 2019 | 800 | 7 | 0 | 0.753 | 2.140 | 1.700 | 1.650 | 9.715 | 0.0026 | 1.259 | 3.543 | 0.0003 | 11.429 | 4396 |  |
| Brassicaceae | *Teesdalia* | *nudicaulis* | 2019 | 800 | 93 | 1 | 0.878 | 1.400 | 0.900 | 0.393 | 0.614 | 0.0003 | 1.556 | 1.577 | 0.0005 | 3.958 | 13510 |  |
| Campanulaceae | *Legousia* | *speculum-veneris* | 2019 | 800 | 97 | 0 | 0.788 | 1.000 | 0.750 | 0.297 | 0.173 | 0.0002 | 1.333 | 1.111 | 0.001 | 2.356 | 14058 |  |
| Caprifoliaceae | *Scabiosa* | *columbaria* | 2019 | 800 | 11 | 0 | 1.186 | 5.863 | 1.925 | 1.750 | 34.129 | 0.0021 | 3.046 | 7.548 | 0.0001 | 41.280 | 19618 |  |
| Caprifoliaceae | *Valerianella* | *dentata* | 2019 | 800 | 83 | 1 | 0.969 | 1.750 | 0.950 | 0.800 | 0.668 | 0.0009 | 1.842 | 2.130 | 0.0013 | 5.223 | 5803 |  |
| Caprifoliaceae | *Valerianella* | *rimosa* | 2019 | 800 | 68 | 14 | 0.608 | 2.000 | 2.000 | 1.537 | 3.043 | 0.0012 | 1.000 | 3.537 | 0.0004 | 12.566 | 10385 |  |
| Caryophyllaceae | *Silene* | *latifolia* | 2020 | 700 | 97 | 2 | 0.756 | 1.516 | 1.199 | 0.850 | 0.830 | 0.0009 | 1.265 | 2.026 | 0.0011 | 5.712 | 6222 |  |
| Caryophyllaceae | *Stellaria* | *media* | 2020 | 700 | 27 | 2 | 0.673 | 1.148 | 1.036 | 0.545 | 0.381 | 0.0004 | 1.108 | 1.430 | 0.001 | 3.733 | 9334 |  |
| Fabaceae | *Lotus* | *corniculatus* | 2019 | 800 | 74 | 3 | 0.722 | 1.240 | 1.036 | 1.279 | 0.469 | 0.0009 | 1.198 | 1.903 | 0.0019 | 4.036 | 4624 |  |
| Fabaceae | *Lupinus* | *polyphyllus* | 2019 | 800 | 52 | 6 | 0.788 | 5.000 | 3.750 | 2.247 | 25.525 | 0.0212 | 1.333 | 9.213 | 0.0008 | 58.905 | 2779 |  |
| Fabaceae | *Medicago* | *sativa* | 2019 | 800 | 95 | 4 | 0.932 | 3.035 | 1.767 | 1.025 | 4.394 | 0.0024 | 1.717 | 3.940 | 0.0005 | 16.845 | 7160 |  |
| Fabaceae | *Onobrychis* | *viciifolia* | 2019 | 800 | 52 | 0 | 0.912 | 4.550 | 2.750 | 2.698 | 13.169 | 0.0178 | 1.655 | 8.259 | 0.0014 | 39.309 | 2208 |  |
| Fabaceae | *Trifolium* | *hybridum* | 2019 | 800 | 99 | 1 | 0.702 | 1.217 | 1.050 | 0.765 | 0.459 | 0.0007 | 1.159 | 1.618 | 0.0015 | 4.013 | 5733 |  |
| Fabaceae | *Trifolium* | *pratense* | 2019, 2020 | 1500 | 75 | 6 | 0.810 | 3.048 | 2.203 | 0.968 | 4.606 | 0.0013 | 1.384 | 4.114 | 0.0003 | 21.097 | 16228 |  |
| Fabaceae | *Trifolium* | *repens* | 2019 | 800 | 89 | 5 | 0.726 | 1.548 | 1.285 | 0.688 | 0.992 | 0.0007 | 1.205 | 1.990 | 0.0007 | 6.247 | 8924 |  |
| Lythraceae | *Lythrum* | *salicaria* | 2020 | 700 | 9 | 6 | 1.010 | 0.400 | 0.200 | 0.308 | 0.005 | 0.0001 | 2.000 | 0.431 | 0.0147 | 0.314 | 4488 |  |
|  |  |  |  |  |  |  |  |  |  |  |  |  |  |  |  |  |  |  |
| **Table S3 (continued):** | | | | | | | | | | | | | | | | | |  |
| Malvaceae | *Malva* | *sylvestris* | 2019 | 800 | 93 | 1 | 0.710 | 2.148 | 1.830 | 1.343 | 11.301 | 0.0055 | 1.173 | 3.376 | 0.0005 | 12.348 | 2245 |  |
| Onagraceae | *Epilobium* | *hirsutum* | 2020 | 685 | 80 | 4 | 1.077 | 0.864 | 0.374 | 0.328 | 0.037 | 0.0001 | 2.314 | 0.926 | 0.0027 | 1.234 | 12335 |  |
| Onagraceae | *Oenothera* | *biennis* | 2020 | 700 | 77 | 6 | 0.943 | 1.626 | 0.928 | 1.100 | 0.555 | 0.0004 | 1.753 | 2.137 | 0.0007 | 4.740 | 11849 |  |
| Papaveraceae | *Papaver* | *argemone* | 2019 | 800 | 71 | 10 | 0.985 | 0.870 | 0.458 | 0.395 | 0.055 | 0.0002 | 1.902 | 0.960 | 0.0027 | 1.250 | 8336 |  |
| Plumbaginaceae | *Armeria* | *maritima* | 2019 | 800 | 7 | 0 | 1.007 | 2.000 | 1.007 | 0.960 | 0.660 | 0.0014 | 1.987 | 2.483 | 0.0021 | 7.918 | 5655 |  |
| Poaceae | *Dactylis* | *glomerata* | 2019 | 800 | 75 | 2 | 1.320 | 4.882 | 1.024 | 0.766 | 1.915 | 0.0008 | 4.768 | 5.274 | 0.0004 | 17.354 | 21692 |  |
| Poaceae | *Elymus* | *repens* | 2020 | 685 | 35 | 8 | 1.306 | 7.221 | 1.598 | 1.057 | 4.585 | 0.0037 | 4.518 | 8.065 | 0.0008 | 40.264 | 10754 |  |
| Poaceae | *Festuca* | *rubra* | 2019 | 800 | 88 | 1 | 1.365 | 6.433 | 1.099 | 0.614 | 2.229 | 0.0012 | 5.851 | 6.770 | 0.0005 | 24.115 | 20095 |  |
| Poaceae | *Lolium* | *perenne* | 2019, 2020 | 1500 | 87 | 12 | 1.295 | 6.260 | 1.448 | 0.779 | 4.126 | 0.002 | 4.324 | 6.824 | 0.0005 | 31.765 | 15882 |  |
| Poaceae | *Poa* | *annua* | 2020 | 685 | 1 | 15 | 1.231 | 2.754 | 0.791 | 0.601 | 0.598 | 0.0003 | 3.481 | 2.992 | 0.0005 | 7.827 | 26090 |  |
| Poaceae | *Poa* | *trivialis* | 2020 | 700 | 33 | 11 | 1.303 | 2.631 | 0.590 | 0.494 | 0.328 | 0.0001 | 4.462 | 2.777 | 0.0003 | 5.420 | 54201 |  |
| Poaceae | *Sorghum* | *bicolor* | 2019 | 800 | 100 | 2 | 0.776 | 4.363 | 3.338 | 3.140 | 12.195 | 0.0132 | 1.307 | 9.603 | 0.0011 | 45.750 | 3466 |  |
| Violaceae | *Viola* | *arvensis* | 2020 | 700 | 46 | 0 | 0.942 | 1.633 | 0.933 | 0.810 | 0.488 | 0.0006 | 1.750 | 2.011 | 0.0012 | 4.789 | 8402 |  |

**⸸ S**eeds were ordered at RH (Rieger-Hofmann, https://www.rieger-hofmann.de), TK (Templiner Kräutergarten, https://templiner-kraeutergarten.de), AS (Asklepios Seeds, https://www.asklepios-seeds.de), PP (Pflanzen-
 Pflanzen, https://www.pflanzen-pflanzen.de), or SC (self-collected in the field, Bavaria, Germany).
**⸸** Seed traits (length, width, height , volume, mass) were available on the CC- BY (Ganhão and Dias 2019) database, the SID (SID Database, 2021) or the LEDA (Kleyer et al. 2008) database.

α Experiments were conducted either in 2019, 2020, or during both years.

β Number of seeds overall fed, numbers < 700 result from limited availability of the respective seeds.

µ Seed surface area was calculated with the formula for elliptical objects for seeds with (V_s_ < 1), and cylindrical objects for seeds with (V_s_ > 1). Variance in dimensions was calculated as: $V_{s}= \sum\frac{{{(x}_{i}- \bar{x})}^{2}}{3} , with x_{1}= \frac{length}{length} , x_{2}= \frac{width}{length} , x_{1}= \frac{height}{length}$ , formula from Bekker et al. (1998).


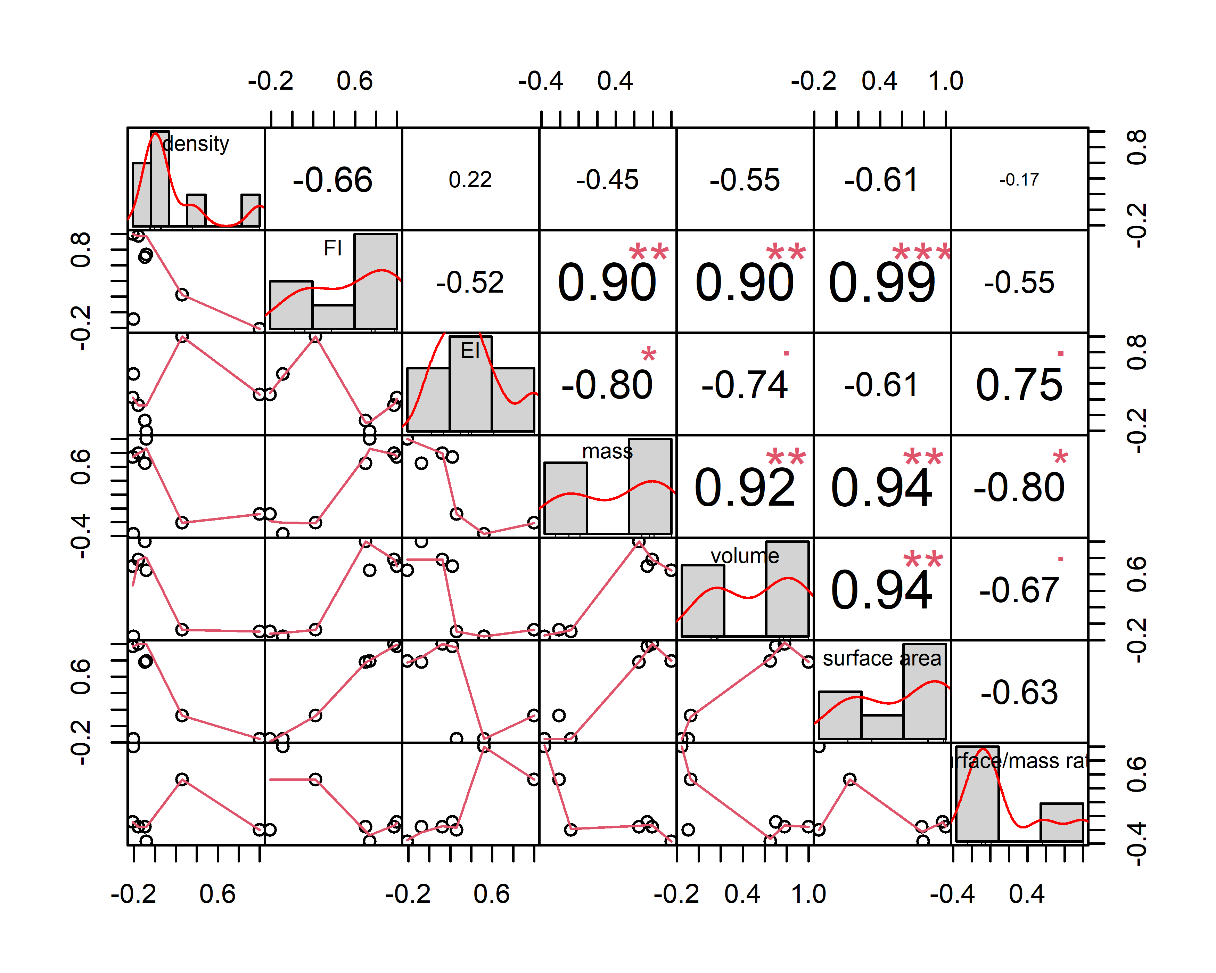


**Fig. S1**: Chart of the pearson correlation matrix of all variables initially considered for statistical modelling (variables with correlation coefficient > 0.7 were excluded from subsequent analysis. The distribution of each variable is shown on the diagonal. On the bottom of the diagonal, the bivariate scatter plots with a fitted line are displayed. On the top of the diagonal, the value of the correlation plus the significance levels are shown as asterisks. p-values (≤ 0.001 ***, ≤ 0.01 **, ≤ 0.05 *, > 0.05 n.s.).


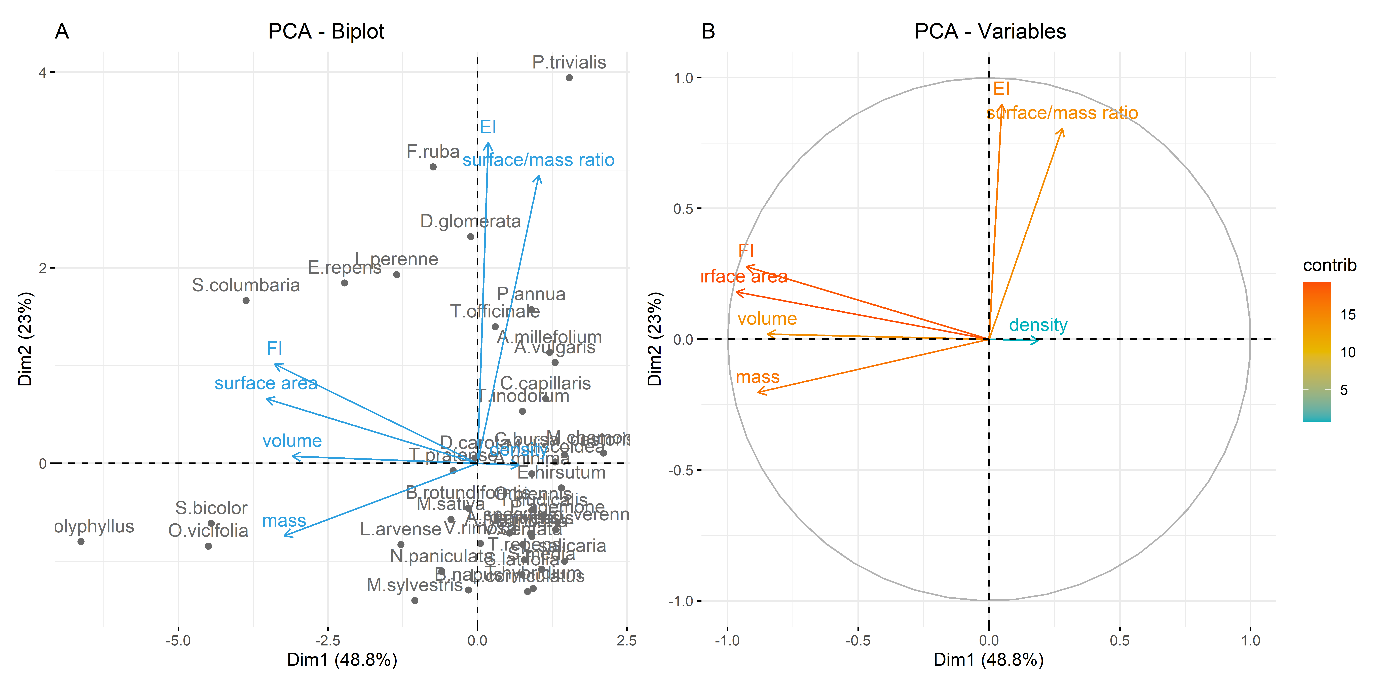


**Fig. S2**: Biplot of individual plant species and all variables initially considered for statistical modelling (A). Graph of variables (B), positive correlated variables point to the same side of the plot. Negative correlated variables point to opposite sides of the graph. We used variables with the highest contribution per dimension (EI, density and surface area), illustrated by the length and color of the arrow, for subsequent analysis.
